# Markers of immune activation: novel biomarkers to predict the early-warning indicator of patients with papillary thyroid carcinoma

**DOI:** 10.1186/s13000-020-00931-1

**Published:** 2020-02-12

**Authors:** Hongsheng Lu, Lihong Zhang, Yuechu Dai, Yanyun Ruan, Xuequan Cao, Xiaobo Cai, Sihan Ruan, Qi Chen

**Affiliations:** 1 Department of pathology, Taizhou Central Hospital (Taizhou University Hospital), Taizhou, Zhejiang, People’s Republic of China; 2 Department of Clinical Laboratory Center, Shaoxing People’s Hospital, Shaoxing, Zhejiang People’s Republic of China; 3 Department of Surgical Oncology, Taizhou Central Hospital (Taizhou University Hospital), Taizhou, Zhejiang People’s Republic of China; 4 Department of Central Laboratory, Taizhou Central Hospital (Taizhou University Hospital), Taizhou, 318000 Zhejiang People’s Republic of China

**Keywords:** Papillary thyroid carcinoma, Thyroid nodules, Lymph node metastasis, Immune activation, Biomarkers, Diagnostic performance

## Abstract

**Background:**

Papillary thyroid carcinoma (PTC) is an indolent tumor that is exploding with increasing thyroid nodules (TN). Environmental carcinogens, lifestyle changes increased the incidence of thyroid carcinoma. With the development of B-ultrasound imaging, more and more thyroid cancer has been found. There has been a debate about whether thyroid cancer is overtreated.

**Methods:**

The expression of T cell subsets and plasma cytokines in 191 patients, including 79 patients with PTC (PTC group), 58 patients with thyroid nodules (TN group) and 54 healthy individuals (HP group) were analyzed by flow cytometry.

**Results:**

High levels of natural killer cells (NK) were detected in PTC and TN groups than in HP group. High activities of CD8^+^HLA-DR^+^ and CD8^+^CD38^+^ showed a gradual upward trend from HP group to PTC group. The rise in the levels of TNF-α in PTC patients’ was evident when compared with HP group. CD8^+^CD38^+^ showed a significant correlation with lymph node metastasis. CD8^+^CD38^+^ co-expression was higher in Nx stage than N_0_ stage, while the proportion of IL-10 was dramatically decreased in the Nx stage.

**Conclusions:**

These results indicated that CD8^+^CD38^+^ might act as a biomarker of PTC lymph node metastasis. The combination of CD8^+^HLA-DR^+^, CD8^+^CD38^+^ and TNF-α can be used as useful biomarkers for the early-warning indicator of PTC.

## Introduction

Thyroid cancer is the most common type of endocrine malignancy and reported an increasing incidence of it worldwide. According to the latest statistics of thyroid cancer incidence in 2018, it accounted for 3.1% of all malignant tumors [1]. Even in the United States, there is an increasing mortality rate associated with thyroid cancer, but is secondary to liver cancer [2]. Among the types of thyroid cancers, Papillary thyroid carcinoma (PTC) is the most common type, which accounted for about 80%. Currently, PTC is considered as an indolent tumor due to its excellent prognosis in majority of the patients [3]. For example, the incidence of thyroid cancer has been increased from 4.56% in 1974–1977 to 14.42% in 2010–2013, with an average annual growth rate of 3.6%, and the incidence of PTC has been increased by an average of 4.4% per year [4].

Surgical resection is a better curative therapy, especially for early differentiated thyroid cancers without local lymph node metastasis [5]. However, lymph node metastasis occurs in patients when the primary PTC is very small, and these patients are associated with poor prognosis. After surgery, there is still certain proportion of patients with recurrence, which is mainly related to the micrometastasis of tumor cells. Some studies have reported the association of micrometastasis in tumors with imbalances in immune function [6, 7]. CD8^+^ T cells represent a subset of T suppressor/cytotoxic cells, and the proportion of CD4^+^/CD8^+^ T cells is decreased, indicating the inhibition of T cell immunity [8]. Philip et al [9] have reported that CD38^+^ T cells were identified as surface markers of some tumor-infiltrating lymphocytes (TILs) and expression of these is associated with chromatin state. Loss of CD38^+^ T cell function is associated with impaired immune responses. CD38 and HLA-DR molecules are transmembrane glycoproteins that are present on immature T and B lymphocytes, and are re-expressed during cellular immune response. CD38 and HLA-DR on CD8^+^ T cells are considered as markers of immune activation. CD8^+^ T cells recognize endogenous antigenic peptides that are presented by MHC class I molecules and are mainly differentiated into cytotoxic T cells (CTLs) after activation [10].

Hence, in this study, the expression levels of CD8^+^CD38^+^, CD8^+^HLA-DR^+^, CD4^+^CD25^+^ T cells, NK cells and B cells (by flow cytometry) among PTC patients (PTC group), thyroid nodule patients (TN group) and normal population (HP group) were measured. Also the expression of plasma cytokines, such as interleukin-2 (IL-2), IL-4, IL-6, IL-10, IFN-γ and tumor necrosis factor-alpha (TNF-α) was explored. Whether the activation of CD8^+^ T cells could act as a novel predictive biomarker for lymph node metastasis of PTC is also studied. Collectively, this study provides guidance regarding to predict the early-warning indicator of PTC, early assessment of thyroid cancer lymph node metastasis.

## Materials and methods

### Ethics statement

This study was approved by the Ethics Committee of Taizhou Central Hospital. All experiments were performed in accordance with approved guidelines. All patients were explained regarding the study protocol and written informed consent was obtained from each patient.

### Patient recruitment

A total of 79 patients with PTC, 58 patients with thyroid nodules and 54 healthy individuals were recruited from the Taizhou Central Hospital between March 2017 and January 2019. The PTC population included 60 females and 19 males, with an age range of 19–72 years. There was no significant difference in age and gender among PTC, TN and HP groups (Table 1). In addition, the patients’ clinical stage, tumor stage and lymph node stage were listed according to the tumor/node/metastasis (TNM) classification [11].

**Table 1:** papillary thyroid carcinoma patients’ demographic and tumor characteristics

	PTC(*n* = 79)	TN((*n* = 58)	HP(*n* = 54)	*p*
Age (year)				0.074
Median	49	49	52	
Range	19–72	14–85	19–77	
Gender				0.210
Male	19	11	18	
Female	60	47	36	
Tumor status				
T_1_	72			
T_2_	5			
T_3_	2			
Nodal status				
N_0_	43			
N_1_	36			
Metastasis status				
M_0_	79			
M_1_	0			

### Samples

Venous blood samples were taken from all subjects in the morning under fasting conditions. Three milliliter peripheral blood was collected from PTC and TN patients and HP population, respectively. EDTA-K2 was added into the blood samples for anticoagulation. The samples were then underwent flow cytometric analysis. The plasma samples were centrifuged for 5 min at 800×g. After centrifugation, the samples were measured by flow cytometry (FCM) using cytometric beads array (CBA) in time.

### Flow cytometric analysis

FCM was used to determine the counts of T, B, NK, and Treg subsets. Cells from peripheral blood monocyte suspension were stained with antibodies by incubating for 30-min in the dark. Peripheral blood mononuclear cells in PBS with 5% heparin were activated by fetal calf serum and were stained for surface markers CD4-FITC, and CD25-APC according to the manufacturer’s instructions. HLA-DR-APC, CD3-PerCP, CD4-FITC, and CD8-PE were added into the second tube. CD38-FITC and CD8+-PE antibodies were added into the third tube. FITC-Mouse-IgG1, PE-Mouse-IgG1, and APC-Mouse-IgG1 were added into the fourth tube. Cells were washed twice during staining and analyzed immediately using BD-FACS AriaII cytometer and FlowJo software (BD Biosciences). Quadrants and box gates were set as isotype controls, and the percentages of CD3^+^CD4^+^, CD3^+^CD8^+^, NK, and B cells, CD4^+^HLA-DR^+^, CD4^+^CD25^+^ and CD8^+^CD38^+^ subsets were then calculated.

### Monoclonal antibodies

Fluorochrome-conjugated monoclonal antibodies (mAbs) used included FITC-Mouse-IgG1 (clone X40), PE-Mouse-IgG1 (clone MOPC-21), APC-Mouse-IgG1 (clone SJ25C1), PerCP-Mouse-IgG1 (clone X40), APC-CD25 (clone 2A3), PerCP-CD4 (clone SK3), FITC-CD8 (clone 2D1), CD19-APC (clone SJ25C1), HLA-DR (clone L243), CD38-APC (clone HB7), CD3-FITC (clone SK7), CD16 + 56-PE (clone B73.1), CD45-PerCP (clone 2D1), CD4-FITC (clone SK3), CD8-PE (clone SK1) (BD Biosciences, San Jose, CA).

### Plasma Th1/Th2 cytokines CBA

FCM was used to measure the concentration of plasma cytokines such as IL-2, IL-4, IL-6, IL-10, IFN-γ and TNF-α. A total of 6 targets including plasma cytokines were detected by Th1/Th2 cytokines CBA (BD Biosciences) using BD-FACS AriaII cytometer. All the experiments were conducted according to the manufacturer’s instructions.

### Statistical analysis

Statistical analysis was performed by using SPSS18.0 software (IBM, Armonk, NY, USA), and GraphPad Prism 6 (GraphPad® Software, Inc., San Diego, CA, USA) was used to generate the graphs. *p* < 0.05 was considered to be statistically significant. Gender and other classification data were analyzed by χ^2^ test.

Independent-samples t-test and Mann-Whitney U-test were used to compare the targets such as CD4^+^, CD8^+^, CD38^+^ and plasma cytokines IL-6, IL-10 and so on among PTC, TN and HP groups. The diagnostic accuracy of diagnostic indicators was compared using the receiver operating characteristic curve (ROC), and the diagnostic accuracy of the indicators was evaluated according to the area under the curve (AUC).

## Results

### NK and CD4^+^CD25^+^ cells were markedly increased in PTC and TN patients

**Fig. 1:**
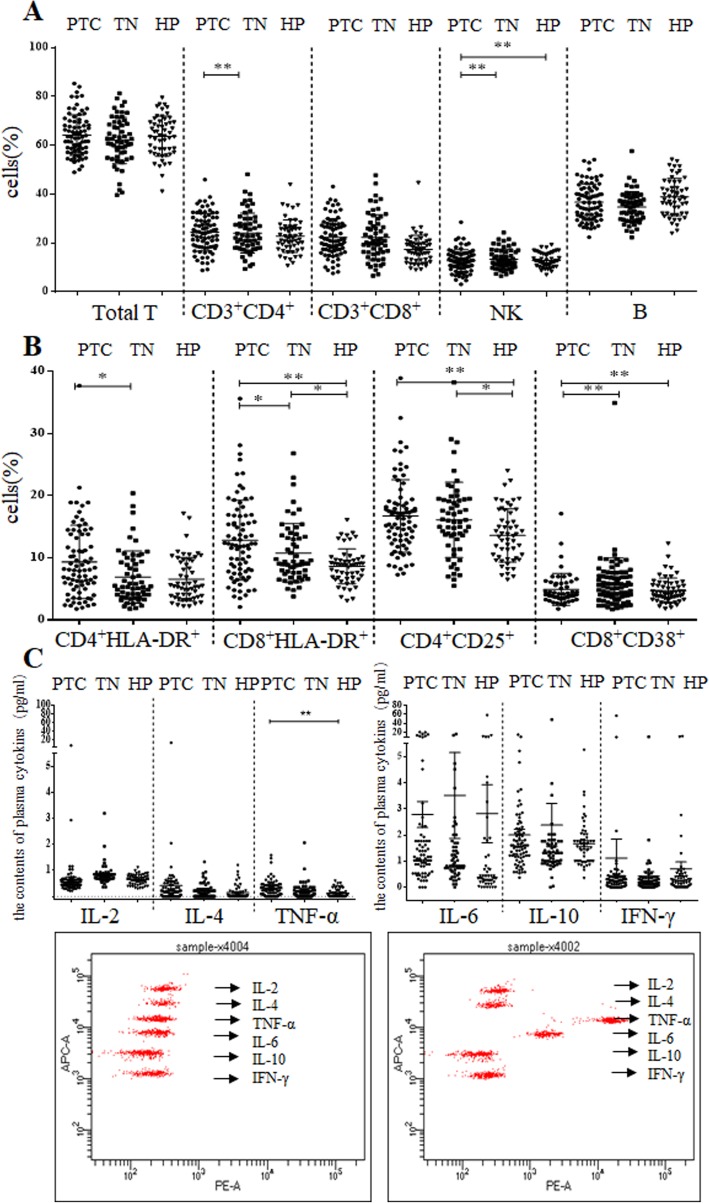
The serum activities of the NK and B cells, T cell subset and plasma cytokines. **a**. The serum activities of the Total T cells, CD3^+^CD4^+^ cells, CD3^+^CD8^+^ cells, NK and B cells among NP, TN, PTC groups. **b**. The serum activities of the CD4^+^HLA-DR^+^, CD8^+^HLA-DR^+^, CD4^+^CD25^+^ and CD8^+^CD38^+^ among NP, TN, PTC groups. **c**. The serum activities of the IL-2, IL-4, IL-6, IL-10, TNF-α and IFN-r amomg NP, TN, PTC groups. * represents significant differences (*p* < 0.05)

Firstly, the expression of T cells, NK cells, B cells, and CD3^+^CD4^+^ cells and CD3^+^CD8^+^ cells from HP, TN and PTC groups were verified (Fig. 1a, b). The results revealed an imbalance in the innate immunity of patients with thyroid disease. Compared with HP group, patients in the TN and PTC had significantly higher expression of NK and CD4^+^CD25^+^ cells (*p* < 0.01), (Table 2). In addition, no significant difference was observed in total T cells, CD3^+^CD4^+^ cells, CD3^+^CD8^+^ cells and B cells among the 3 groups (*p* > 0.05).

**Table 2:** The changes of lymphocyte subsets and plasma cytokines among PTC, TN and HP

Parameters	PTC	TN	HP	*p1*	*p2*	*p3*
Total T(%)	64.16 ± 8.31	61.95 ± 9.44	63.67 ± 8.23	0.142	0.752	0.294
CD3^+^CD4^+^(%)	36.66 ± 7.42	34.67 ± 6.31	38.79 ± 7.60	0.109	0.093	0.003
CD3^+^CD8^+^(%)	24.36 ± 7.67	23.97 ± 8.24	22.72 ± 6.70	0.763	0.223	0.388
CD4^+^HLA-DR^+^(%)	5.89 ± 4.06	4.75 ± 1.98	4.93 ± 2.57	0.036	0.085	0.758
CD8^+^ HLA-DR^+^(%)	12.84 ± 6.41	10.75 ± 4.72	8.62 ± 2.82	0.019	0.000	0.028
CD4^+^ CD25^+^(%)	16.74 ± 5.83	16.10 ± 6.04	13.62 ± 4.27	0.500	0.002	0.018
CD8^+^ CD38^+^(%)	9.34 ± 5.89	6.92 ± 4.19	6.57 ± 3.51	0.004	0.001	0.711
NK(%)	22.21 ± 7.62	22.27 ± 9.28	17.32 ± 5.80	0.962	0.000	0.001
B(%)	12.70 ± 4.49	13.30 ± 4.08	12.83 ± 2.81	0.379	0.849	0.530
IL-2(pg/ml)	0.65 ± 0.89	0.83 ± 0.40	0.64 ± 0.19	0.084	0.981	0.109
IL-4(pg/ml)	0.41 ± 1.61	0.18 ± 0.28	0.14 ± 0.27	0.219	0.150	0.824
IL-6(pg/ml)	2.79 ± 4.45	3.52 ± 12.55	2.82 ± 8.20	0.626	0.984	0.669
IL-10(pg/ml)	2.01 ± 1.97	2.38 ± 6.30	1.67 ± 0.87	0.557	0.614	0.314
TNF-α (pg/ml)	0.30 ± 0.32	0.20 ± 0.32	0.13 ± 0.16	0.069	0.001	0.162
IFN-γ (pg/ml)	1.12 ± 6.48	0.42 ± 1.34	0.70 ± 1.98	0.356	0.590	0.732

*p1*: TPC and TN, *p2*:TPC and HP, *p3*:TN and HP

### Differential expression of lymphocyte activation related indexes in PTC, TN and HP patients

The results of FCM showed no significant difference in the expression of CD4^+^HLA-DR^+^ between PTC and HP groups (*p* > 0.05). The expression of CD4^+^HLA-DR^+^ showed no significant difference in both TN group and HP group (*p* > 0.05), and a significant difference between PTC and TN groups was observed (*p* < 0.05). However, the activation of CD8^+^HLA-DR^+^ T cells was very obvious. CD8^+^HLA-DR^+^ T cells from HP to PTC, and with disease progression, the expression ratio was significantly increased (*p* < 0.05). These results indicated that CD8^+^CD38^+^ cells were mainly expressed in patients with TN and PTC (*p* < 0.05) (Fig. 1,b Table 2).

### The expression of plasma cytokines among HP, TN and PTC groups

The expression of plasma cytokines of IL-2, IL-4, IL-6, IL-10 and IFN-γ showed no significant changes among the 3 groups (*p* > 0.05). In contrast, TNF-α was weakly expressed in HP and TN groups, but the expression was enhanced in the presence of PTC, showing an association with lymph node metastasis (Fig. 1c).

### Effects of different lymph node stages on T cell subsets and plasma cytokines

**Fig. 2:**
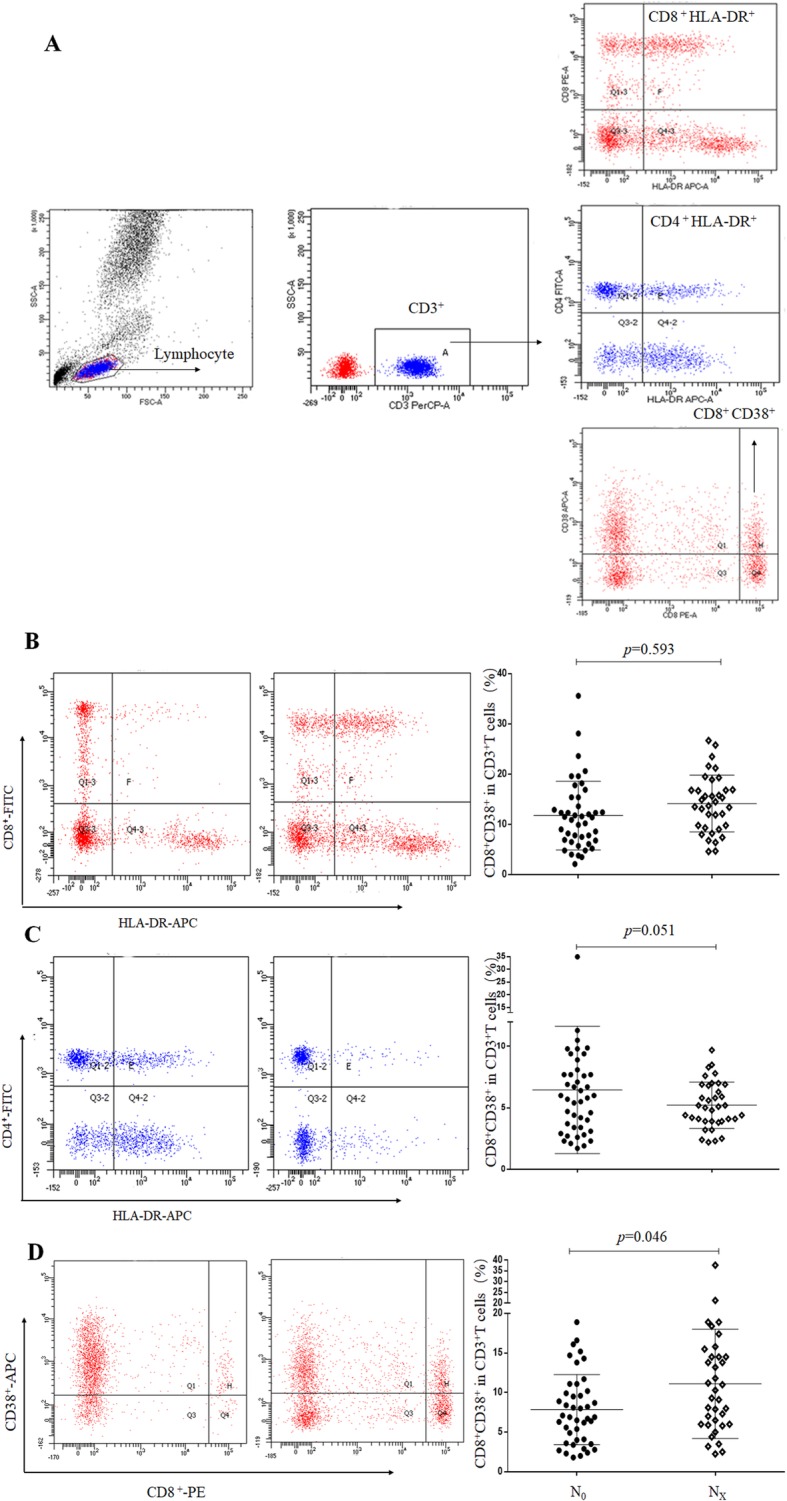
T subsets in peripheral blood of PTC patients with N_0_ and N_x_ stage. **a**. The CD3^+^, CD4^+^, CD8^+^ and CD38^+^ expression detected by flow cytometry. **b**. CD8^+^HLA-DR^+^ expression on peripheral CD3^+^ T cells. **c**. CD4^+^HLA-DR^+^ expression on peripheral CD3^+^ T cells. **d**. CD8^+^CD38^+^ expression on peripheral CD3^+^ T cells. *p* < 0.05 represents a significant difference

**Fig. 3:**
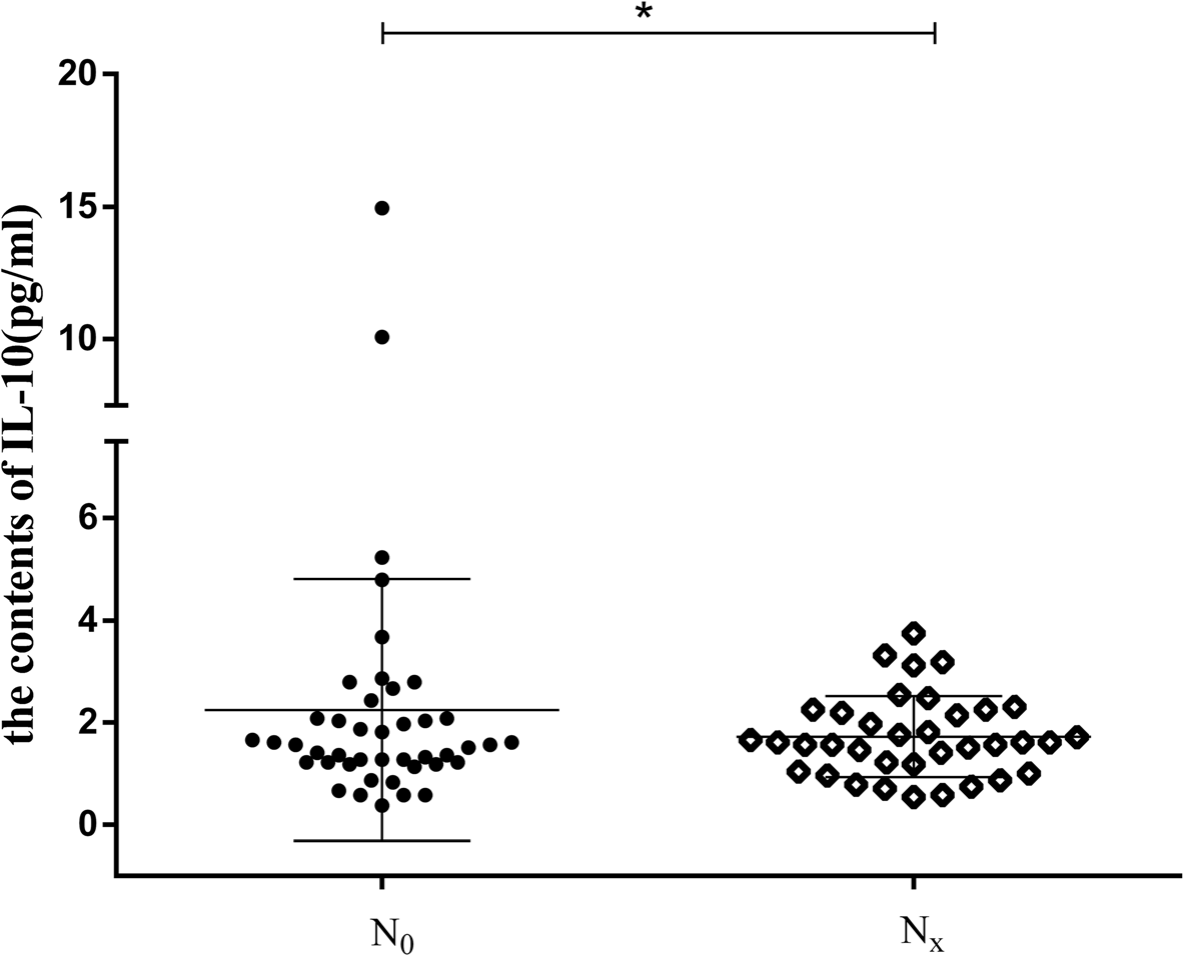
The contents of IL-10 in different lymph node stages. * represents significant differences (*p* < 0.05)

To determine the prognostic value of T cell subsets in PTC patients (Fig. 2a), FCM was used to analyze the effects of T cell subsets on different lymph node stages (N_0_ and N_x_). The results showed that different lymph node stages demonstrated no change in the expression of CD4^+^HLA-DR^+^ and CD8^+^HLA-DR^+^ (Fig. 2b, c), and so the release of TH1/TH2 cytokines was not obvious. In contrast, tumor tissues with high lymph node stage showed more CD8^+^CD38^+^ co-expression than at low lymph node stage (Fig. 2d). In addition, the contents of IL-10 were dramatically decreased in the Nx stage when compared to patients with N_0_ stage of PTC patients (Fig. 3), indicating the existence of adaptive immunity in patients with lymph node metastasis. Furthermore, PTC group with lymph node metastasis showed more CD8^+^CD38^+^ coexpression than those in the TN group (Fig. 1b). These results further indicated that increased CD8^+^CD38^+^ coexpression was a novel predictor of lymph node metastasis in patients with PTC.

### The relationship between immune activation indicators, cytokines and diagnostic performance of PTC

**Fig. 4:**
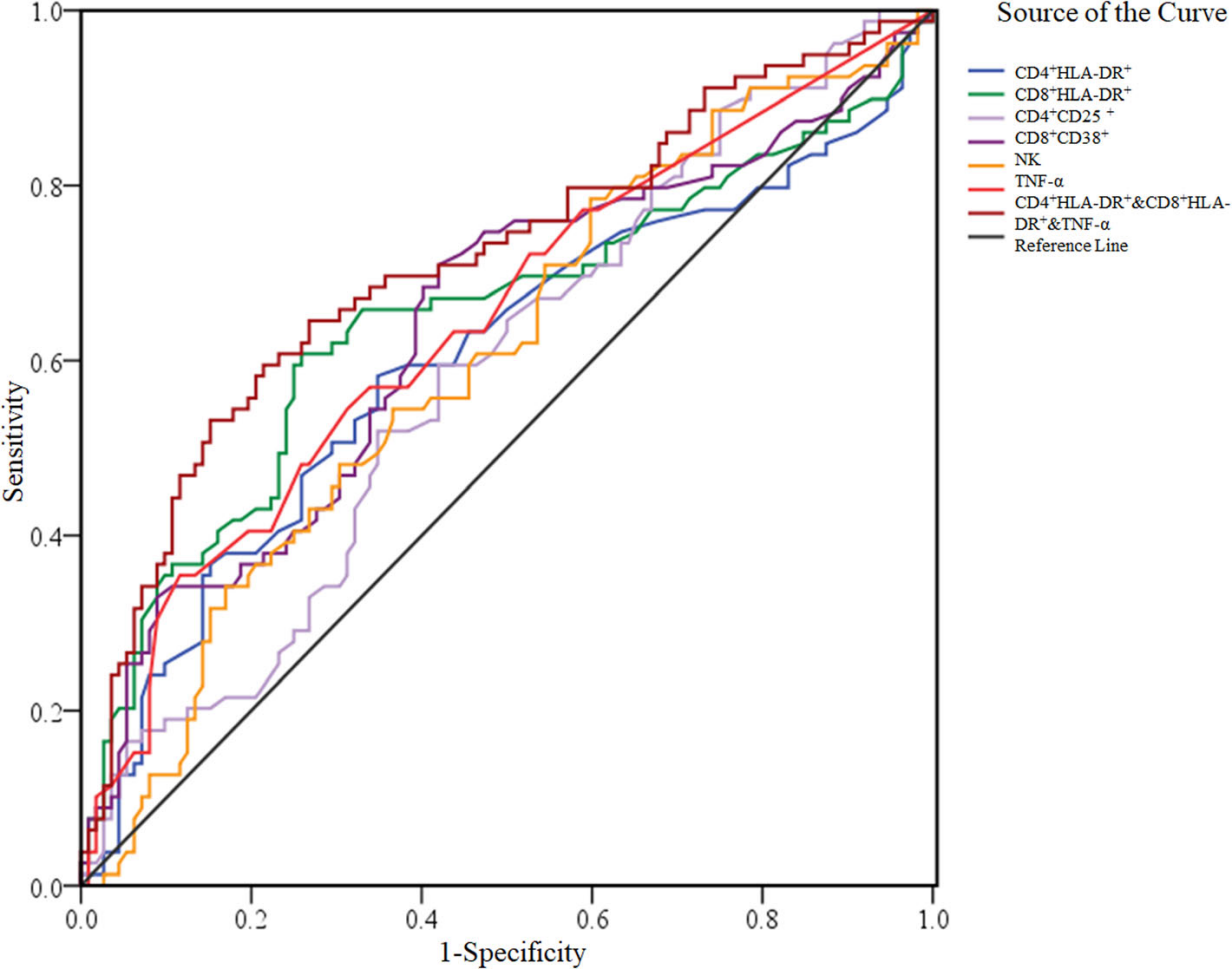
The ROC curve of immune activation related indicators and cytokines

Clinical diagnosis of thyroid cancer was taken as the gold standard, and the ROC curve of immune activation related indicators and cytokines was drawn. The sensitivity was ordinate, 1-specificity was abscissa, and the ROC diagrams of CD4^+^HLA-DR^+^, CD8^+^HLA-DR^+^, CD4^+^CD25^+^, CD8^+^CD38^+^, NK, and TNF-α were plotted. The AUC of the ROC of these was compared. The AUC was 0.601, 0.648, 0.587, 0.637, 0.602 and 0.643, respectively (Table 3, Fig. 4). The AUC of CD8^+^HLA-DR^+^, CD8^+^CD38^+^ and TNF-α combination was 0.713.

**Table 3:** The area under the curve with the gold standard as clinical diagnosis of thyroid cancer

Variables	AUC	*p*	Asymptotic 95% Confidence Interval	
			Lower Bound	Upper Bound
CD4^+^HLA-DR^+^	0.601	0.017	0.516	0.686
CD8^+^HLA-DR^+^	0.648	0.000	0.564	0.732
CD4^+^CD25^+^	0.587	0.041	0.505	0.668
CD8^+^CD38^+^	0.637	0.001	0.555	0.719
NK	0.602	0.016	0.521	0.683
TNF-α	0.643	0.001	0.563	0.724
CD4 + HLA-DR + & CD8 + HLA-DR + & TNF-α	0.713	0.000	0.637	0.790

## Discussion

With the increasing use of techniques such as thyroid ultrasound and fine-needle aspiration, the incidence of PTC has been exploding in recent years [12]. The high prevalence of TN constituted as an enormous reservoir of potential cancer [13]. However, is the incidence of thyroid cancer truly increased? Indeed, some environmental carcinogens, lifestyle changes and more sensitive diagnostic techniques are related to the increased incidence of thyroid cancer, and overdiagnosis still existed [14]. Therefore, in order to better understand the cause of increased incidence rate of thyroid cancer, molecular markers are used to avoid overtreatment.

Tumor cell invasion and metastasis is a complex process, and is a major cause of death in cancer patients [15]. Micrometastasis in tumors is associated with imbalances in immune function and the effect of immune function on lymph node micrometastasis has also been detected in cancer patients. Wen et al. [16] have already demonstrated that low expression of CD4^+^/CD8^+^ is associated with micrometastasis in the lymph nodes of colorectal cancer patients. In addition, T-cell activation in NSCLC tissues was significantly higher for lymph node metastasis [17]. Indeed, T cell immunity acts as an essential factor in immunosuppressive pathway, making it an interested feature in future prognosis of thyroid cancer. Increased CD8^+^ CD38^+^ and CD8^+^ HLA-DR^+^ cells by antigen-reactive T cells has been reported in a number of viral infections and diseases, including HIV-infected individuals, kidney recipients or breast ductal carcinoma [18]. The results of the present study revealed that the activities of CD8^+^HLA-DR^+^ and CD8^+^CD38^+^ cells showed a significant increase in TN and PTC groups (Fig. 1b), indicating the destruction of balance of T cells in immune system. CD38 as a surface antigen is an activation marker of T cells, and HLA-DR acts as a classic marker for lymphocyte activation. There are very few studies till date that have reported on the correlation of CD38^+^ T cells HLA-DR^+^ and thyroid cancer. CD8^+^CD38^+^ showed significant correlation with lymph node metastasis. We speculated that the activities of CD8^+^CD38^+^ cells are associated with micrometastasis in lymph nodes. In our study, the expression of CD38^+^ on T cells showed significant correlation with lymph node stage in PTC patients (Fig. 2d). CD8^+^CD38^+^ coexpression was higher in Nx stage than in N_0_ stage (Fig. 2d).

Normally, the number and proportion of T cells are relatively stable under normal circumstances. In lymphocyte lines, activated lymphocytes re-express high levels of CD38 and HLA-DR. Binding of CD38 to competitive monoclonal antibodies mediates changes in TNF-α, and IL-10 [19]. A dramatic increase in the expression of TNF-α and a marked decrease in the expression of IL-10 in the peripheral blood of PTC patients in Nx stage were observed (Fig. 4). A rise was mostly evident in the levels of TNF-α in PTC patients when compared with HP group.

CD8^+^ cytotoxic T-cell affects the severity of the disease by cellular immunity [20]. CD4^+^ helper T cells are critical regulators of immune responses [21]. Immunosuppression often occurs in cancer patients, and T lymphocyte subsets are often disordered in the peripheral blood of cancer patients. A decreased expression of CD4^+^ cells is usually present, causing immune escape of tumor cells. In contrast, increased proportion of CD8^+^ cells is associated with cellular immune damage. In our study, no significant differences in CD3^+^CD4^+^ cells among HP, TN and PTC groups were found, and high expression of NK cells was detected in TN and PTC groups, which was inconsistent with the results of previous studies [22]. We speculated that an imbalance in innate immunity during the early stage of thyroid disease occurred when clearing the tumor cells. A significantly increased expression of T lymphocytes was observed in PTC patients. There were no significant differences in CD3^+^CD4^+^ cells and CD3^+^CD8^+^ cells among the 3 groups (Fig. 1). However, a rising proportion of CD3^+^CD8^+^ cells still existed, with a proportion of 24.36 ± 7.67 and 22.72 ± 6.70 in PTC and HP groups, respectively (Fig. 1b). This rise was mostly evident in CD8^+^HLA-DR^+^ cells in PTC patients but not in CD4^+^HLA-DR^+^ cells (Fig. 1b), indicating that a proportion of CD8^+^ cells was positively correlated with the severity of the disease.

In conclusion, CD8^+^CD38^+^ cells reflect changes in immunological status. CD8^+^CD38^+^ cells might act as a novel predictor of lymph node metastasis in patients with PTC. The combination of CD8^+^HLA-DR^+^, CD8^+^CD38^+^ and TNF-α can be used as useful biomarkers for the early-warning indicator of PTC.

## Data Availability

All data for this study are presented in the manuscript.
